# Effects of typical and atypical antipsychotic drugs on gene expression profiles in the liver of schizophrenia subjects

**DOI:** 10.1186/1471-244X-9-57

**Published:** 2009-09-16

**Authors:** Kwang H Choi, Brandon W Higgs, Serge Weis, Jonathan Song, Ida C Llenos, Jeannette R Dulay, Robert H Yolken, Maree J Webster

**Affiliations:** 1 Stanley Laboratory of Brain Research, Rockville, MD 20850, USA; 2 Elashoff Consulting, Redwood City, CA 94065, USA; 3 Departments of Psychiatry and Pathology, Uniformed Services University of the Health Sciences, Bethesda, MD, 20814, USA; 4 Stanley Laboratory of Developmental Neurovirology, Johns Hopkins University, School of Medicine, 600 North Wolfe Street, Blalock 1105, Baltimore, MD 21287, USA

## Abstract

**Background:**

Although much progress has been made on antipsychotic drug development, precise mechanisms behind the action of typical and atypical antipsychotics are poorly understood.

**Methods:**

We performed genome-wide expression profiling to study effects of typical antipsychotics and atypical antipsychotics in the postmortem liver of schizophrenia patients using microarrays (Affymetrix U133 plus2.0). We classified the subjects into typical antipsychotics (n = 24) or atypical antipsychotics (n = 26) based on their medication history, and compared gene expression profiles with unaffected controls (n = 34). We further analyzed individual antipsychotic effects on gene expression by sub-classifying the subjects into four major antipsychotic groups including haloperidol, phenothiazines, olanzapine and risperidone.

**Results:**

Typical antipsychotics affected genes associated with nuclear protein, stress responses and phosphorylation, whereas atypical antipsychotics affected genes associated with golgi/endoplasmic reticulum and cytoplasm transport. Comparison between typical antipsychotics and atypical antipsychotics further identified genes associated with lipid metabolism and mitochondrial function. Analyses on individual antipsychotics revealed a set of genes (151 transcripts, FDR adjusted p < 0.05) that are differentially regulated by four antipsychotics, particularly by phenothiazines, in the liver of schizophrenia patients.

**Conclusion:**

Typical antipsychotics and atypical antipsychotics affect different genes and biological function in the liver. Typical antipsychotic phenothiazines exert robust effects on gene expression in the liver that may lead to liver toxicity. The genes found in the current study may benefit antipsychotic drug development with better therapeutic and side effect profiles.

## Background

Differential therapeutic and side effects of typical antipsychotic (AP) and atypical AP drugs in schizophrenia have been documented [1,2]. Typical APs such as haloperidol and phenothiazines induce elevation of serum prolactin, extrapyramidal symptoms and tardive dyskinesia, whereas atypical APs such as clozapine and olanzapine induce metabolic syndromes and elevation of liver enzyme levels [3-6]. However, precise mechanisms underlying the effects of typical and atypical AP drugs on gene expression in the postmortem liver of schizophrenia patients are poorly understood. Unlike typical APs, atypical APs appear to increase liver enzyme function although they induce less hepatotoxicity [7-9]. The atypical AP drug clozapine induces a metabolic syndrome by down-regulating cytochrome P450 (CYP450) isozymes and by causing an accumulation of fatty acids in the liver [10]. However, certain typical APs, including chlorpromazine and haloperidol, may also decrease the activity of CYP450 isozymes in the liver of rats [11,12]. While the effects of APs on liver function have been confirmed in individuals with schizophrenia, additional pharmacological and clinical factors could also contribute to altered liver function [13]. Interestingly, a study suggested that the metabolic alterations leading to oxidative stress in the liver of schizophrenia patients may actually be linked to the disease process itself [14].

Previous postmortem brain studies identified gene expression changes in metabolism-related pathways in schizophrenia [15,16]. However, other studies found that AP medication may act to compensate for the underlying pathological deficits in the metabolic pathways in schizophrenia [17,18]. For instance, genes involved in lipid metabolism and cellular signaling are altered in the mice brains by chronic AP treatment [19], suggesting that the metabolic abnormalities may also be a function of the AP medication. In schizophrenia, abnormal myelination and oligodendrocytes have been described [20] and both typical and atypical APs may regulate the expression of genes associated with lipid biosynthesis and myelination in cultured human glioma cells [21]. Interestingly, these genes are controlled by the sterol regulatory element-binding protein (SREBP) transcription factors. Thus, SREBP-mediated increase in glial cell lipogenesis could be one of the potential mechanisms behind the AP medication. Also, genes associated with cell cycle, intracellular signaling, oxidative stress and metabolic functions are altered in the lymphocytes of schizophrenia patients compared to normal controls [22]. However, this study identified the schizophrenia subjects as medicated, minimally medicated and un-medicated, so that it is difficult to interpret which AP class affected those genes. In the human liver tissues, typical APs and atypical APs may mediate different functions leading to liver toxicity in schizophrenia patients who had taken typical APs [23]. However, atypical AP treatment may increase levels of liver enzymes such as alanine aminotransfeaminotransferase (ALT), aspartate aminotransferase (AST), gamma-glutamyl transferase (GGT), and alkaline phosphotase (ALP) [7]. Taken together, these studies suggest that typical APs may cause liver toxicity whereas atypical APs may regulate liver enzyme functions. To our knowledge, the effects of different APs on genome-wide expression profiles in the postmortem liver of schizophrenia patients have not been reported.

Given the various effects of APs on genes and biological functions in different tissues, we investigated the effects of typical APs and atypical APs on gene expression profiles in the postmortem liver of schizophrenia patients. We classified the schizophrenia subjects into either typical AP group or atypical AP group based on their medication history from one or two years prior to death, and compared gene expression profiles with unaffected controls. We further analyzed individual AP medication effects on gene expression by sub-classifying the subjects into four major AP drug groups including phenothiazines, haloperidol, olanzapine and risperidone.

## Methods

### Postmortem liver tissues

Postmortem liver tissues were obtained from the Stanley Medical Research Institute (SMRI). The details of the postmortem tissue collection have been described previously [24]. Information on medication was taken from the clinical and the medical records, reviewed in each case. The postmortem tissues were collected between 1995 and 2005 during the period when the use of typical AP medication was decreasing and the use of atypical AP medication was increasing. Thus, individual patients at the time of death were being treated with typical or atypical AP drugs. For quality of the tissue, exclusion criteria included: age>65 years, poor quality RNA, and significant structural pathology of the liver on postmortem examination. Samples were matched for age, gender, race, pH and total RNA quality. Total RNA quality was determined by the Bioanalyzer 2100 electrophoresis system (Agilent Technologies, Foster City, CA, USA) using RNA Integrity Number (RIN) as previously described [25]. The schizophrenia subjects were classified into two groups: those who had taken predominantly typical APs (phenothiazines, thioxanthenes, butyrophenones, and diphenylbutylpiperidines) and those who had taken predominantly atypical APs (clozapine, risperidone, olanzapine, quetiapine, and aripiprazole). The brain collection protocol was reviewed and approved by the SMRI. Detailed information on ethical approval can be found at .

### Microarray experiment

Frozen postmortem liver tissue was homogenized in Trizol (Invitrogen, Carlsbad, CA), and the RNA was separated with chloroform and high-speed centrifugation. RNA was precipitated with isopropyl alcohol and washed with 70% ethanol and the pellets of RNA were resuspended in DEPC water [25]. An additional step of RNeasy column purification (Qiagen, Valencia, CA) was added to increase the efficiency of RNA quantification and purity. A genome-wide expression microarray experiment was carried out using the Affymetrix chips (HG-U133 plus2.0, 54,675 transcripts) at the Microarray Core Facility of the Johns Hopkins University (Baltimore, MD). Microarray data including cel files, normalized data and demographic information can be found at the Stanley Online Genomics Database (, Study id: 19).

### Quality control of microarrays

The Affymetrix Microarray Analysis Suite 5.0 (MAS 5.0) expression values were calculated based on scaling to a target intensity of 100, then transformed by log_2 _(x+20). Absent/Present calls were computed using the MAS 5.0 algorithm. The Absent/Present call rate was used for gene filtering prior to the data analysis. All analysis was conducted using the R statistical environment (R Development Core Team (2007). R: A language and environment for statistical computing. R Foundation for Statistical Computing, Vienna, Austria). For the quality control (QC) analysis, several primary QC metrics were used including: scale factor, percent present, number of probes with perfect match>mis-match, 5'/3' GAPDH, 5'/3' Actin and average correlation. For each metric, we computed the distribution of the metric across the samples within each study. Although no hard cutoffs were applied for each of the QC metrics, we examined the distribution of the metrics to determine whether samples appeared to be outliers as described previously [26-29].

### Demographic and clinical variable analyses

Each demographic and clinical variable was assessed using regression analysis. The percentage of regulated probes in each variable was calculated based on the criteria of significance (p < 0.001 and fold change >1.3). For the comparison of effect sizes, all demographics were analyzed using two levels. Continuous variables and ordered categorical variables were cut at median values for the regression analysis. Demographic factors were assessed using all samples including those from the unaffected controls, and the typical and atypical AP groups. Schizophrenia-specific variables were analyzed only in schizophrenia cases to avoid the confounding of demographic effects and disease effects. The following demographic and clinical variables were considered for all subjects: age, gender, postmortem interval (PMI), body mass index (BMI), pH, mRNA quality, heavy alcohol use, heavy drug use and rate of death. The following clinical variables were considered for the subjects with schizophrenia: global severity of disease, suicide status, exacerbation of disease at the time of death, insight, and duration of illness. Global severity of disease is an estimate of the severity of illness for the entire course of illness. All schizophrenia patients were rated against others. This assessment includes both symptom severity and social disability. Exacerbation of disease is an estimate of whether the person's symptoms were getting worse at the time of death. Heavy drug and alcohol use reflect substance abuse in the past and at time of death. Insight is an assessment of the individual's awareness of his/her illness. The assessment is based on the medical records or from the family about whether the individual voluntarily sought treatment and complied with medication.

### Antipsychotic medication analysis

Demographic and clinical variable analyses revealed several confounding variables affecting expression of a significant number of genes in the liver (refrigerator time, PMI, rate of death, RNA quality, heavy drug use, heavy alcohol use, gender and suicide). Therefore, these confounding variables were adjusted for AP class analysis in a series of linear regression models, one model for each gene, including typical or atypical AP drugs and eight confounding variables as covariates and gene expression intensity (log2 scale) as the dependent variable. The criteria of significance for each gene were FC >1.3 and p < 0.001 after adjusting for the confounding variables.

Following the AP class comparisons, individual AP drug effects on gene expression in the liver were investigated. Based on the recent medication history (one or two years prior to death), the subjects were sub-classified into four individual AP drug groups including phenothiazines (n = 12), haloperidol (n = 9), olanzapine (n = 11) and risperidone (n = 10). Schizophrenia subjects (n = 8) who had taken both typical and atypical AP drugs during this period were excluded. Individual AP drug comparisons were performed using a single factor ANOVA to identify genes that are differentially expressed among the AP drug groups (FDR adjusted p < 0.05). The results from individual AP drug analysis were compared with the results from AP class comparisons (multiple regression analyses) to identify common set of genes between the two analyses.

### Bioinformatic mappings

The NCBI's Database for Annotation, Visualization and Integrated Discovery (DAVID, ) was used as the standard source for gene annotation information [30]. In the DAVID annotation system, the Fisher's Exact test was used to measure the gene-enrichment in annotation terms. The primary fields extracted from the DAVID include Entrez Gene ID, gene symbol and gene summary. Additional annotations included gene product mappings to the Gene Ontology Consortium (GO) for GO terms.

### Quantitative PCR

Total RNA was extracted from the postmortem liver tissue, and the quality of RNA was assessed with the Bioanalyzer 2100 (Agilent, Foster City, CA). RNA was further purified with the PureLink Micro to Midi Total RNA Purification System (Invitrogen, Carlsbad, CA), and cDNA was synthesized with RT-PCR using oligo dT primers. Using a 384-well format with the ABI Prism 7900HT real-time detector, 1 μl aliquots of QuantiTect SYBR primer (20×), 10 μl QPCR PCR Master mix (Applied Biosystems, Foster City, CA), and 10 μl diluted cDNA were mixed together and pipetted into single wells of the qPCR plate. Water was added instead of cDNA in the no template controls (NTC) for each gene tested. Thermo cycle conditions were: (1) 1 cycle for 2 min at 50°C, (2) 1 cycle for 15 min at 95°C, and (3) 40 cycles for 15 sec at 95°C and 1 min at 60°C and fluorescence was measured during the 60°C step for each cycle as recommended by the manufacturer. Target genes include ATP-binding cassette, sub-family G, member 5 (ABCG5, NM_022436, QT00023415), androgen receptor 1 (AR1, NM_005650, QT00076615), CCAAT/enhancer binding protein, alpha (CEBPA, (NM_004364, QT00203357), cytochrome P450, family 51, subfamily A, polypeptide 1 (CYP51A1, NM_000786, QT00055790), cytochrome P450, family 7, subfamily A, polypeptide 1 (CYP7A1, NM_000780, QT00001085), FOS-like antigen 2 (FOSL2, NM_005253, QT01000881), interleukin 1 receptor antagonist (IL1RN, NM_173843, QT01002918) and superoxide dismutase 2 (SOD2, NM_000636, QT01008693). Three endogenous control genes were selected for the qPCR experiment: β-2 microglobulin (B2M, NM_004048, QT00088935), glyceraldehyde-3-phosphate dehydrogenase (GAPDH, NM_002046, QT01192646) and β-actin (ACTB, NM_001101, QT00095431). Polymerase chain reactions were quantified by the relative ΔΔCt method using the SDS2.2 software (Applied Biosystems, Foster City, CA). An average Ct value for each sample from the triplicates of that sample was calculated for each gene. Geometric mean of three endogenous control genes (B2M, ACTB and GAPDH) was used to normalize the data for each gene of interest. The normalized values for each gene of interest in the typical AP class were expressed as fold change (FC) as compared to the atypical AP class.

## Results

Table 1 shows a summary of subject characteristics with demographic and clinical variables. Demographic variables such as age, gender, race and BMI are matched between the controls, the typical AP and the atypical AP group. Both typical and atypical AP groups had a higher incidence of suicide and a longer PMI compared to the control group. Subsequent analysis on individual variables revealed that suicide and PMI affected expression of a significant number of genes. Thus, these variables were adjusted in the AP medication analysis using the multiple regression models.

**Table 1:** A summary of subject characteristics.

	**Unaffected Control**	**Typical AP**	**Atypical AP**
No. of Subjects	34	24	26
Age	45.9 ± 1.8	47.1 ± 2.2	42.8 ± 2.4
Gender (Male)	73%	63%	65%
Race (White)	94%	92%	89%
pH	6.4 ± 0.1	6.4 ± 0.1	6.5 ± 0.1
PMI	27.2 ± 2.5	36.3 ± 3.7	37.1 ± 4.7
BMI	29.2 ± 1.5	29.6 ± 1.3	30.5 ± 1.5
Heavy Drug Use	9%	9%	12%
Heavy Alcohol Use	9%	14%	8%
Suicide	0%	24%	27%

PMI: postmortem interval, BMI: body mass index

**Figure 1:**
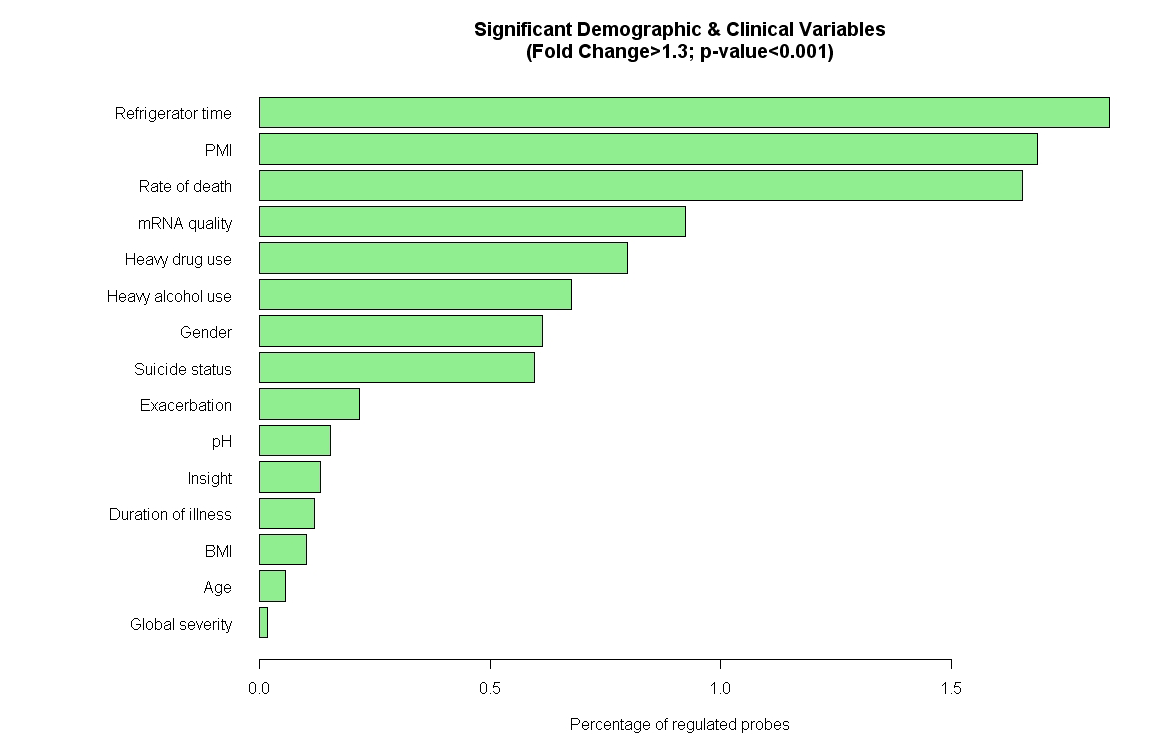
**Individual demographic and clinical variable analyses**. Three variables including refrigerator time, PMI and rate of death affected more than 1% of the transcripts in the liver (fold change >1.3 and p < 0.001). Other variables such as mRNA quality, heavy drug use, heavy alcohol use, gender and suicide status affected the expression levels in the range of 0.5-1%.

Demographic and clinical variable analyses identified potential confounding variables affecting the expression of a significant number of transcripts in the postmortem liver (Figure 1). Three variables including refrigerator time, PMI and rate of death affected more than 1% of the transcripts based on the significant criteria of fold change >1.3 and p < 0.001. Other variables including RNA quality, heavy drug use, heavy alcohol use, gender and suicide affected the expression levels in the range of 0.5-1%.

For the analysis of typical AP and atypical AP effects, eight confounding variables (refrigerator time, PMI, rate of death, mRNA quality, heavy drug use, heavy alcohol use, gender and suicide) were adjusted on a gene-by-gene level with multiple regression models in order to compute adjusted p-values and fold changes. Among these variables, suicide was a schizophrenia-specific variable and thus, this variable was adjusted only in the schizophrenia subjects (typical AP and atypical AP groups). Table 2 shows a summary of fold change (FC) and p-values for the genes in each comparison including the typical AP vs. the controls, the atypical AP vs. the controls, and the typical AP vs. the atypical AP. The comparison between the typical AP and the control group revealed 426 transcripts (p < 0.001 and FDR of 4%). Among the 426 transcripts, 103 transcripts showed FC >2, indicating robust effects of typical APs on gene expression in the liver. The comparison between the atypical AP and the control group revealed 75 transcripts (p < 0.001 and FDR of 24%). Among the 75 transcripts, only 4 transcripts show FC >2, indicating modest effects of atypical APs compared to typical APs. The comparison between the typical APs and the atypical APs revealed 160 transcripts (p < 0.001 and FDR of 11%) and 17 transcripts showed FC >2. See Additional Files 1, 2 and 3 for a list of significant transcripts (FC>1.3 and p < 0.001) in each comparison.

**Table 2:** A summary of fold changes and p-values for the transcripts (p < 0.01) in comparison between the typical AP vs. the controls, the atypical AP vs. the controls, and the typical AP vs. the atypical AP.

**Fold change**	**P-value**								
	**<1e-04**			**1e-04-0.001**			**0.001-0.01**		
	**Typical**	**Atypical**	**T vs. AT**	**Typical**	**Atypical**	**T vs. AT**	**Typical**	**Atypical**	**T vs. AT**
1-1.5	19	7	9	124	46	67	607	371	518
1.5 - 2	69	4	11	111	14	56	212	55	200
2-2.5	31	3	1	22	0	12	28	5	24
>2.5	35	0	0	15	1	4	12	1	13
Total	154	14	21	272	61	139	859	432	755
									
Cumulative Total	154	14	21	426	75	160	1285	507	915
FDR (%)	1	14	10	4	24	11	14	35	19

Typical: Typical AP group vs. control group; Atypical: Atypical AP group vs. control group; T vs. AT: Typical AP group vs. atypical AP group; FDR: false discovery rate

Following the gene-level analysis, we examined the biological functions of these genes using the DAVID functional annotation. Table 3 shows the biological functions overrepresented in each comparison. For example, genes associated with nuclear protein (p = 3.75E-07), response to stress (p = 4.49E-06) and phosphorylation (p = 1.13E-05) are overrepresented in the comparison between the typical AP and the controls. Genes associated with golgi/endoplasmic reticulum (p = 6.19E-08) and transport function (p = 2.86E-07) are overrepresented in the comparison between the atypical AP and the controls. A comparison between the typical AP and the atypical AP further identified the genes associated with lipid metabolism (p = 9.59E-05), membrane-bound organelle (p = 3.03E-04) and mitochondrion (p = 3.66E-04).

**Table 3:** Significant biological terms in each comparison between typical AP vs. control, atypical AP vs. control, and typical AP vs. atypical AP.

**Comparison**	**Category**	**Term**	**Count**	**%**	**P-Value**
Typical vs. Ctrl	SP_PIR_KEYWORDS	Nuclear protein	70	21%	3.75E-07
Typical vs. Ctrl	GOTERM_BP_ALL	Response to stress	39	12%	4.49E-06
Typical vs. Ctrl	SP_PIR_KEYWORDS	Phosphorylation	46	14%	1.13E-05
Typical vs. Ctrl	INTERPRO_NAME	Basic-leucine zipper (bZIP) transcription factor	8	2%	1.46E-05
Typical vs. Ctrl	GOTERM_MF_ALL	Protein binding	97	29%	1.56E-05
Atypical vs. Ctrl	SP_PIR_KEYWORDS	ER-golgi transport	6	11%	6.19E-08
Atypical vs. Ctrl	GOTERM_CC_ALL	Cytoplasm	26	46%	7.84E-08
Atypical vs. Ctrl	GOTERM_BP_ALL	ER to Golgi vesicle-mediated transport	6	11%	2.86E-07
Atypical vs. Ctrl	SP_PIR_KEYWORDS	Endoplasmic reticulum	9	16%	1.96E-06
Atypical vs. Ctrl	GOTERM_BP_ALL	Golgi vesicle transport	6	11%	2.12E-06
Typical vs. Atypical	GOTERM_BP_ALL	Cellular lipid metabolism	13	10%	9.59E-05
Typical vs. Atypical	GOTERM_BP_ALL	Lipid biosynthesis	9	7%	1.14E-04
Typical vs. Atypical	GOTERM_BP_ALL	Lipid metabolism	14	10%	2.34E-04
Typical vs. Atypical	GOTERM_CC_ALL	Intracellular membrane-bound organelle	54	40%	3.03E-04
Typical vs. Atypical	GOTERM_CC_ALL	Mitochondrion	14	10%	3.66E-04

A set of significant genes (FC>1.3 and p < 0.001) in each comparison was used in the DAVID functional annotation analyses.

**Figure 2:**
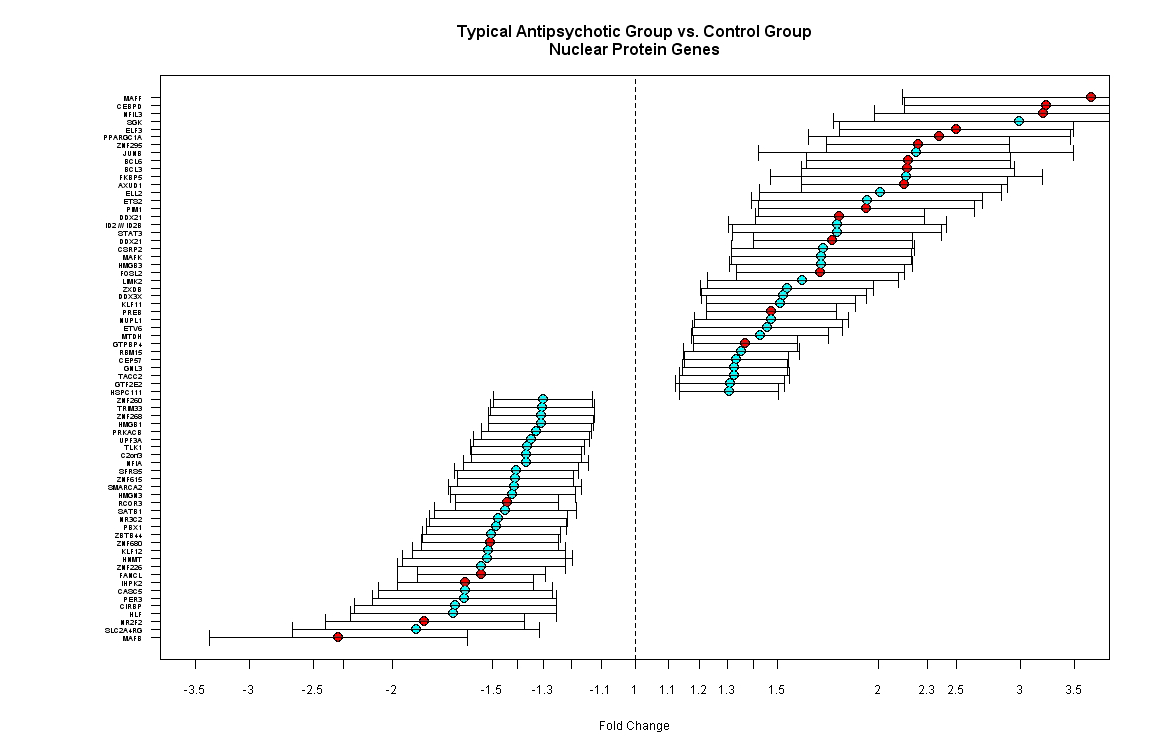
**Genes associated with the nuclear protein function are differentially regulated in the typical AP group compared to the control group**. Each gene is plotted with fold change and 95% confidence intervals. Green: p < 0.001 and red: p < 0.0001

We identified a set of genes significantly associated with specific biological function from the functional annotation. For instance, genes associated with the nuclear protein (FC>1.3 and p < 0.001) were differentially expressed in the typical AP group compared to the controls (Figure 2). Individual genes with fold changes and 95% confidence intervals show that approximately half of the genes are up-regulated and the other half are down-regulated in the nuclear protein category. These genes include many transcription factors and DNA binding proteins that are critical for regulating a cascade of gene expression events in the nucleus of cells.

**Figure 3:**
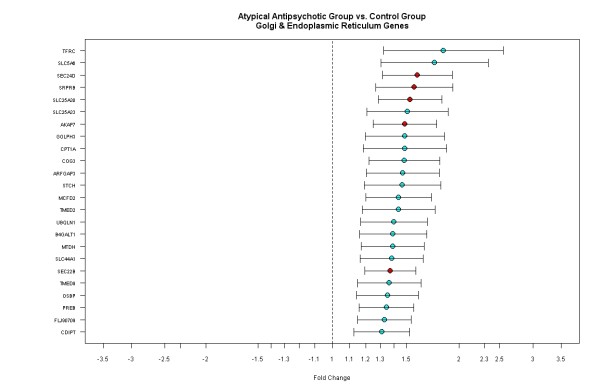
**Genes associated with the golgi/endoplasmic reticulum transport are up-regulated in the atypical AP group compared to the control group**. Each gene is plotted with fold change and 95% confidence intervals. Green: p < 0.001 and red: p < 0.0001

Figure 3 illustrates the genes associated with the golgi/endoplasmic reticulum are consistently up-regulated in the atypical AP group compared to the controls. However, most of the genes show moderate fold changes between 1.3 and 2 compared to the fold changes observed between the typical AP group and the controls. Increased gene expression associated with the golgi/endoplasmic reticulum suggest that atypical APs affect post-translational modifications, rather than the genes involved in direct transcriptional modifications in the nucleus of the cells.

**Figure 4:**
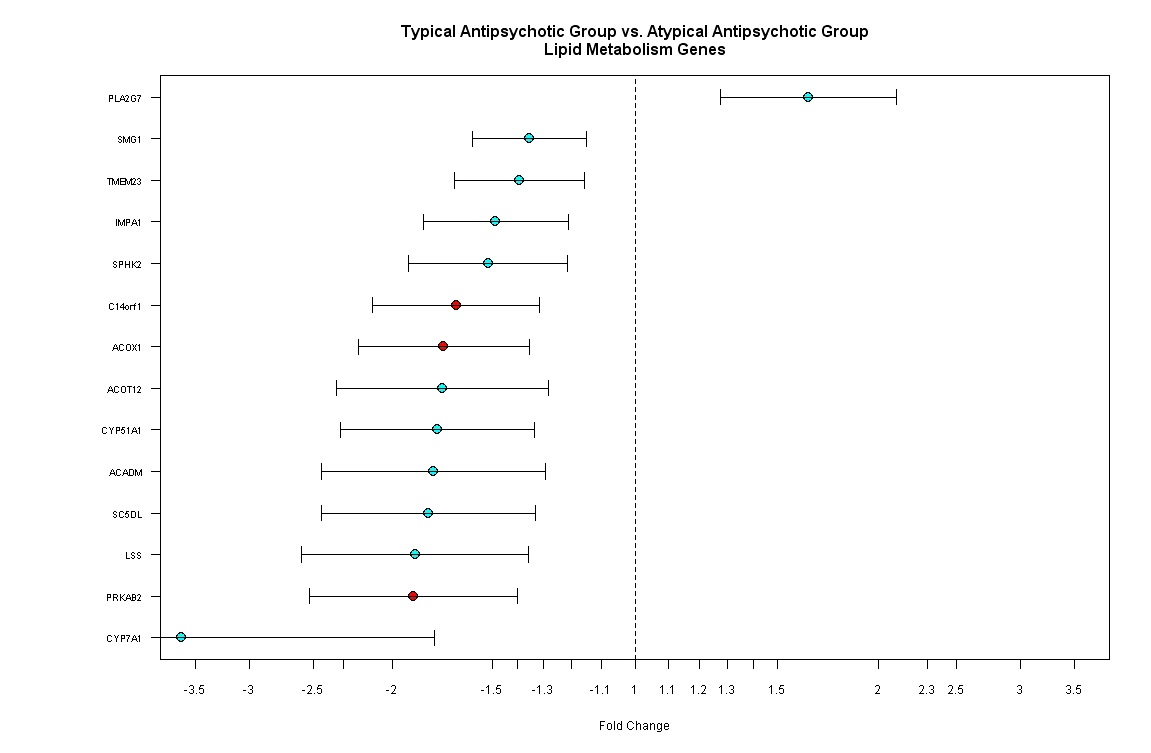
**Genes associated with the lipid metabolism are down-regulated in the typical AP group compared to the atypical AP group**. Each gene is plotted with fold change and 95% confidence intervals. Green: p < 0.001 and red: p < 0.0001

Figure 4 shows the genes associated with the lipid metabolism are consistently down-regulated in the typical AP group compared to the atypical AP group. Two CYP450 isozymes, CYP7A1 and CYP51A1, also show down-regulation in the typical AP group as compared to the atypical AP group. Differential effects of typical APs and atypical APs on lipid biosynthesis and metabolism may provide further evidences for the metabolism-related syndrome that has been observed with atypical APs [18,19,31,32].

**Figure 5:**
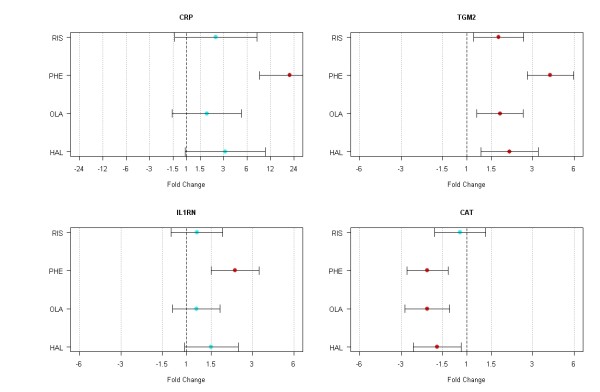
**Effects of individual AP drugs on gene expression are shown with fold change and 95% confidence intervals**. These genes show differential expression profiles in the liver by the individual AP drugs. Values are expressed as fold changes compared to the unaffected controls. Red: significant from the controls (FDR p < 0.05). RIS, risperidone; PHE, phenothiazines; OLA, olanzapine; HAL, haloperidol.

Following the AP class analyses, we analyzed individual AP drug effects on gene expression in the liver. Individual AP drug analysis including haloperidol, phenothiazines, olanzapine, risperidone and the unaffected controls revealed 158 transcripts (FDR p < 0.05) that are differentially regulated among the four AP drug groups. We then compared this result with the previous results from AP class comparisons (typical AP vs. control and atypical AP vs. control). Among the 158 transcripts, we identified 151 transcripts that are common in the typical AP class comparison and 20 transcripts that are common in the atypical AP class comparison. This confirms that typical APs, not atypical APs, exert robust effects on gene expression in the liver. Among those 151 transcripts, 26 transcripts are associated with response to stress based on the functional annotation analysis (adj. p = 0.001, fold enrichment = 3.21). Figure 5 illustrates four example genes that are differentially regulated by individual AP drugs. For instance, C-reactive protein (CRP) expression (FDR p = 0.0002) and interleukin receptor 1 antagonist (IL1RN) expression (FDR p = 0.0004) are selectively increased by the phenothiazines. In contrast, transglutaminase 2 (TGM2) expression is increased by all four AP drugs as compared to the controls (FDR p < 0.0001). A catalase (CAT) gene expression is decreased by phenothiazines, haloperidol, and olanzapine, but not by risperidone (FDR p < 0.01). Detailed information on the 151 genes is shown in Additional File 4.

**Figure 6:**
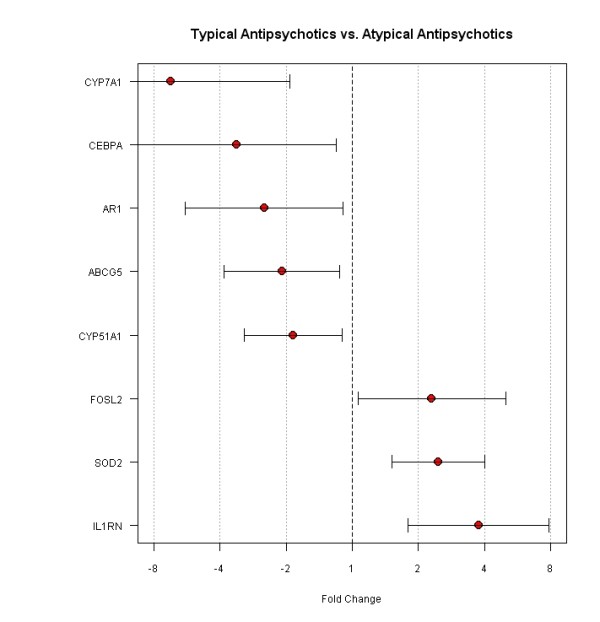
**The qPCR validation of genes differentially expressed between the typical AP group and the atypical AP group based on the microarray data analysis**. Normalized values for each gene in the typical AP group were expressed as fold change as compared to the atypical AP group. Each gene is shown with fold change and 95% confidence intervals.

Following the microarray analysis, we performed quantitative PCR to validate a set of genes that are differentially expressed between the typical AP and the atypical AP groups. Figure 6 demonstrates that 5 genes, CYP7A1, CEBPA, AR1, ABCG5 and CYP51A1, are down-regulated and 3 genes, FOSL2, SOD2 and IL1RN, are up-regulated in the typical AP group compared to the atypical AP group. The magnitude of the fold changes are similar to the fold changes observed in the microarray data analysis, confirming the consistency between these two different gene expression assays.

## Discussion

It is well accepted that typical APs and atypical APs mediate differential therapeutic and side effects in individuals who are taking AP medications. Most previous studies have focused on the effects of typical APs and atypical APs on gene expression and on drug metabolism using animal models. Recent studies have investigated the effects of the APs in the postmortem brains using gene expression microarrays [33,34]. However, to our knowledge, none has reported the effects of APs on global gene expression profiles in the postmortem liver of schizophrenia patients. Based on the previous studies on liver function, it is likely that atypical APs have fewer side effects and less liver toxicity than typical APs [35,36] and these differences may be partially due to the differential gene expression pattern induced by two different classes of AP drugs.

### Effects of typical antipsychotics on gene expression

We found that typical APs affected the genes associated with nuclear protein, response to stress and phosphorylation in the liver (Table 3). The genes associated with nuclear protein include many transcription factors and DNA binding proteins that are crucial to regulating expression of other genes in the nucleus of cells. The typical AP haloperidol has been shown to induce DNA methylation changes in the brain and peripheral tissue of rats [37]. Another typical APs phenothiazines may contribute to liver toxicity [23], extrapyramidal side effects [38] and chromosomal DNA damage [39]. Thus, these studies suggest that typical APs may regulate biological functions related to nuclear protein and stress responses in the liver of schizophrenia patients.

### Effects of atypical antipsychotics on gene expression

In contrast, atypical APs affected genes associated with the golgi apparatus/endoplasmic reticulum in the liver. The genes associated with this category were consistently up-regulated, suggesting that atypical APs may regulate transport mechanisms in the cytoplasm rather than affecting gene expression cascades in the nucleus of cells. We found that the genes associated with cytoplasmic function are up-regulated in the atypical AP group (Additional file 5). Previous animal studies reported that chronic administration of atypical APs, in contrast to typical APs, does not cause toxic effects in the liver [40]. However, the atypical AP clozapine does induce a metabolic syndrome including weight gain, glucose tolerance and insulin sensitivity via alteration of glucose metabolism in rats [41]. Our results suggest that there are clear differences between the typical APs and the atypical APs on gene expression profiles in the liver of schizophrenia patients, consistent with the previous animal studies [19,42].

### Comparison between typical antipsychotics and atypical antipsychotics

Comparison between the typical APs and the atypical APs revealed that genes associated with lipid metabolism and biosynthesis are differentially regulated. Two CYP450 isozyme genes, CYP51A1 and CYP7A1, were down-regulated and these changes were confirmed by the qPCR. Although previous studies reported the significance of CYP450 systems in AP drug metabolism, the role of these two isozymes have not been reported. The significance of the metabolic syndrome in schizophrenia, particularly the potential side effects of atypical APs on lipid metabolism, has been described previously [43,44]. For example, the typical AP drug haloperidol reduced expression of CYP450 genes in the liver of rats [45]. Many APs are metabolized by the CYP450 isozymes and also the enzyme activities are also regulated by the APs [46,47]. Therefore, a subset of CYP450 isozymes may have different responses to typical APs or atypical APs in the liver of schizophrenia patients.

Another biological function between the typical APs and the atypical APs was mitochondrial function with 11 genes down-regulated and 3 genes up-regulated (Additional file 6). A study reported mitochondrial dysfunction in schizophrenia [48] and mitochondrial genes may also be affected by AP medication. For example, the APs induced changes in mitochondria-related genes in postmortem brains of schizophrenia [49]. The authors suggested that this was a medication effect rather than the disease itself because the brains of AP-free schizophrenia cases did not show similar effects on the mitochondrial genes. Moreover, typical and atypical AP drugs exert different effects on mitochondrial function in the rat liver and these differences may provide a possible link to extrapyramidal symptoms observed in patients taking typical APs [50]. The typical AP, thioridazine, also interacts with the inner membrane of mitochondria, acquiring antioxidant activity toward processes with potential implications in apoptosis [51]. Taken together, these results suggest that APs affect the genes associated with mitochondrial function in the brain and in the liver.

There were 20 genes common between two comparisons (typical AP vs. control and atypical AP vs. control) based on the significance criteria (FC>1.3 and p < 0.001). These genes include enzymes such as transglutaminase 2 (TGM2), nicotinamide N-methyltransferase (NNMT), and inositol(myo)-1(or 4)-monophosphatase 2 (IMPA2). Therefore, these enzymes may be involved in common metabolic pathways affected by both typical AP and atypical AP classes in the liver of schizophrenia patients. Although we only investigated gene expression profiles in the liver, it is possible that these genes may be affected by both typical APs and atypical APs in other tissues. Since both typical AP and atypical AP drugs improve positive symptoms of schizophrenia [18], the common genes found between two comparisons may provide clues to similar therapeutic and side effect profiles.

### Effects of individual antipsychotic drugs on gene expression

Based on individual AP drug comparisons, we identified 158 transcripts that are differentially expressed among four AP drugs (FDR adjusted p < 0.05). Among the four AP drugs compared, the phenothiazines affected most of the genes, and a subset of those genes (n = 26) were associated with stress responses (adjusted p = 0.001, fold enrichment = 3.21). This indicates that phenothiazines (chlorpromazine, fluphenazine and thioridazine) with similar chemical structure produce robust effects on gene expression that could contribute to liver toxicity [23], extrapyramidal side effects [38] and even chromosomal DNA damage [39] observed with phenothiazines. For instance, we found that expression of C-reactive protein (CRP), a marker of inflammation, was dramatically increased by the phenothiazines (FC 21, FDR-adjusted p = 0.0002). This corroborates a recent study that reported serum CRP levels are increased more in schizophrenia patients who had taken typical APs as compared to those who had taken atypical APs [52]. This also suggests that the phenothiazines affect the immune system and may cause oxidative stress in the peripheral tissues.

### qPCR validation

Quantitative PCR has become a standard for validating gene expression changes identified in microarray studies [53,54]. We selected a set of genes differentially expressed between the typical AP and the atypical AP classes, and validated the gene expression using qPCR. The CCAAT/enhancer binding protein alpha (CEBPA) gene encodes a basic leucine zipper motif (bZIP) transcription factor that binds to the promoter and modulates the expression of the leptin gene, a protein that plays an important role in body weight homeostasis. The typical AP chlorpromazine has been shown to increase insulin sensitivity in the liver by attenuating insulin and leptin signaling pathways [55]. It is interesting that one of the main side effects of atypical APs is weight gain and dysfunction of the CEBPA gene may contribute to this side effect. The ATP-binding cassette (ABCG5) gene is a member of the superfamily of ATP-binding cassette (ABC) transporters. ABC proteins transport various molecules across extra- and intra-cellular membranes and are expressed in liver tissue. Polymorphisms in the ABC gene are associated with treatment resistance in schizophrenia and response to the atypical AP drug risperidone in schizophrenia [56,57]. These studies suggest that the ABC genes may play a role in responses to different APs in schizophrenia.

Among the up-regulated genes, FOS-like antigen 2 (FOSL2) encodes leucine zipper proteins that can dimerize with proteins of the JUN family, thereby forming the transcription factor complex AP-1. Chronic treatment with APs induces long-lasting expression of the FOS and JUN family genes and AP-1 complex in the rat prefrontal cortex [58]. Although APs have been shown to regulate the expression of FOS and JUN family genes in the rat brain [58-60], the effect of typical APs and atypical APs on these genes in the liver have not been reported. The superoxide dismutase 2 (SOD2) gene encodes a mitochondrial protein that forms a homotetramer and binds one manganese ion per subunit. The functions of the SOD2 protein include binding to the superoxide byproducts of oxidative phosphorylation and converting them to hydrogen peroxide and diatomic oxygen. While chronic administration of the typical AP chlorpromazine decreases SOD enzyme activity in the brain and in erythrocytes [61], the effects in the liver are not known. The interleukin 1 receptor antagonist (IL1RN) gene inhibits the activities of interleukin 1, and modulates a variety of interleukin 1-related immune and inflammatory responses. Genes associated with the immune and inflammatory responses have been reported in schizophrenia [62,63]. Functional polymorphisms in the interleukin genes have been reported in schizophrenia [64] and therefore, interleukin genes may be promising candidates for research in schizophrenia.

### Strength of the current study

It is well known that gene expression studies using postmortem tissues of psychiatric patients are challenging [65-68]. Disease-specific effects are often hindered by issues such as relatively small sample size, small effect size and heterogeneity of symptoms in the patient population [69]. Also, clinical information available on each patient is often incomplete, and so unknown clinical covariates may either confound or confuse many of the gene expression findings [26,28]. Therefore, appropriate statistical adjustment using available information is critical in order to improve inferences in determining candidate genes and the biology behind psychiatric disorders. However, most previous studies have not accounted for these issues or made adequate adjustments using appropriate statistical methods. In this study, we first analyzed the demographic and clinical variables, in order to identify potential confounding effects on gene expression. We then performed the multiple regression analyses to adjust the confounding variables on a gene-by-gene level to obtain adjusted p-values and fold changes for the analysis of AP medication effects.

Previous studies reported that gene expression fold changes between unaffected controls and psychiatric patients are relatively small, and statistical significance is relatively weak, especially after adjusting for multiple confounding variables [27]. Compared to the postmortem brain tissues, we found that the gene expression changes are quite robust in the postmortem liver tissues. This may be due to the direct and profound effects of APs on gene expression in the liver as compared to the confounding medication and disease effects found in the brain. Also, the robust effects observed in the liver may be due to the cell-type homogeneity of the liver compared to the brain, in which several cell types are present in any given region.

## Limitations

Although the current study investigated the effects of AP medications on gene expression profiles in the postmortem liver of schizophrenia patients, caution is required when interpreting the results. Clinical information available from each patient is often incomplete and AP medication effects are confounded by heterogeneous medication regimen. For instance, some patients had a mixed medication history with prescriptions for both typical and atypical AP drugs during the course of the illness. However, the current analysis revealed that the typical AP class, not the atypical AP class, exerts robust gene expression changes in the liver of schizophrenia patients. In order to address individual AP drug effects on gene expression, we sub-classified the schizophrenia subjects into 4 AP groups based on their recent medication history from one or two years prior to death. Although it was not possible to obtain complete medication history and information on medication adherence from each patient, we attempted to identify genes and biological functions that are affected by those four AP drugs including phenothiazines, haloperidol, olanzapine and risperidone in the liver. Due to the limitations inherent to the postmortem tissues of psychiatric patients, the current study should be considered as exploratory rather than conclusive. Further animal and cell culture studies with controlled medication regimen are necessary to extend the findings from the current study.

## Conclusion

Typical AP and atypical AP medication affect different genes and biological functions in the liver of schizophrenia patients. The typical AP phenothiazines produced robust effects on gene expression and a subset of those genes are associated with stress responses in the liver. Thus, the genes and the pathways identified in the current study may provide important clues to the differential therapeutic and side effects mediated by two different classes of APs. Moreover, the current findings may provide useful information for developing novel APs with better therapeutic and side effect profiles.

## Competing interests

The authors declare that they have no competing interests.

## Authors' contributions

KC, BH and SW conception and design, analysis and interpretation of data, drafting the manuscript. JS, IL and JD acquisition of data, analysis and interpretation of data. RY and MW interpretation of data, revisions of the manuscript.

## Pre-publication history

The pre-publication history for this paper can be accessed here:



## Supplementary Material

Additional file 1 **Typical AP vs. Ctrl**. A list of significant genes in the comparison between typical AP group and unaffected control group (FC>1.3 and p < 0.001). Click here for file

Additional file 2 **Atypical AP vs. Ctrl**. A list of significant genes in the comparison between atypical AP group and unaffected control group (FC>1.3, p < 0.001). Click here for file

Additional file 3 **Typical AP vs. Atypical AP**. A list of significant genes in the comparison between typical AP group and atypical AP group (FC>1.3, p < 0.001). Click here for file

Additional file 4 **Individual AP drug effects**. A list of significant genes (n = 151) in the comparisons between individual AP drugs including haloperidol, phenothiazines, olanzapine, and risperidone. (FDR p < 0.05). Click here for file

Additional file 5 **Cytoplasm genes**. Genes associated with the cytoplasm function in atypical AP group compared to unaffected control group. Each gene is plotted with fold change and 95% confidence intervals. Green: p < 0.001 and red: p < 0.0001 Click here for file

Additional file 6 **Mitochondria genes**. Genes associated with the mitochondrial function in typical AP group compared to atypical AP group. Each gene is plotted with fold change and 95% confidence intervals. Green: p < 0.001 and red: p < 0.0001 Click here for file
