# Supplementary figures and images for: Effects of typical and atypical antipsychotic drugs on gene expression profiles in the liver of schizophrenia subjects
Source: BMC Psychiatry. 2009 Sep 16;9:57. doi: 10.1186/1471-244X-9-57 (PMC2749837; doi:10.1186/1471-244X-9-57)

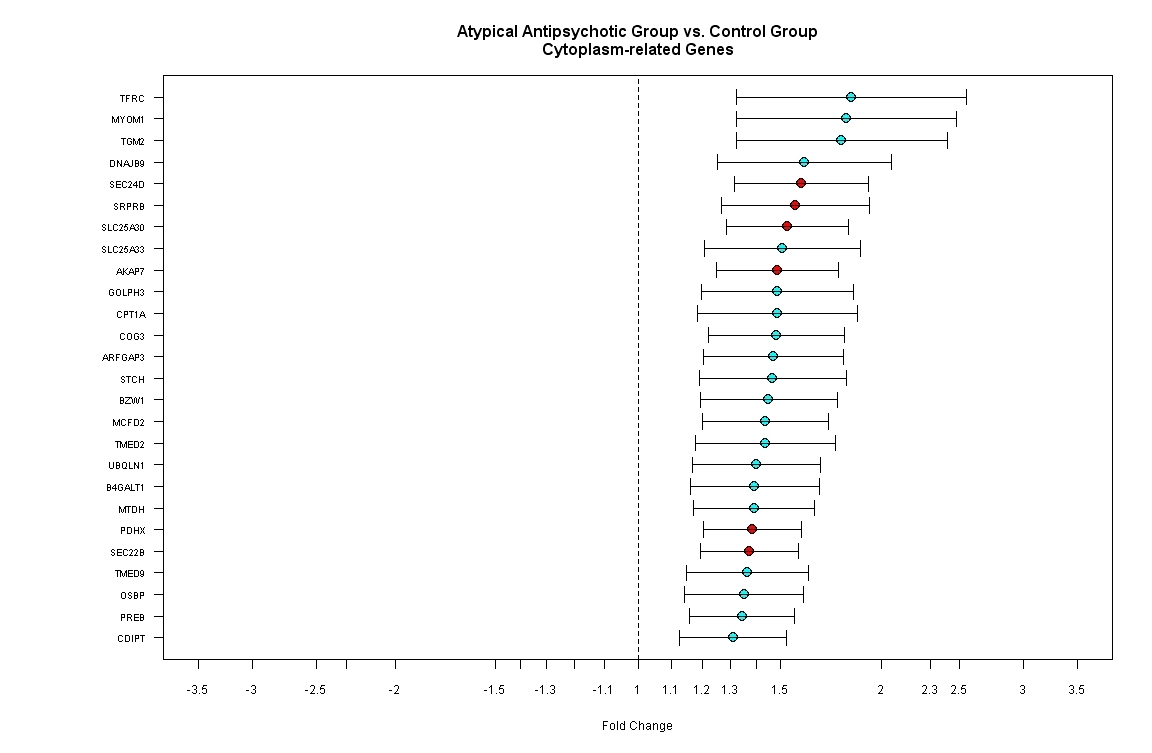

Supplement: Additional file 5 — Cytoplasm genes. Genes associated with the cytoplasm function in atypical AP group compared to unaffected control group. Each gene is plotted with fold change and 95% confidence intervals. Green: p < 0.001 and red: p < 0.0001 [file 1471-244X-9-57-S5.ZIP › additional_file5.jpg]

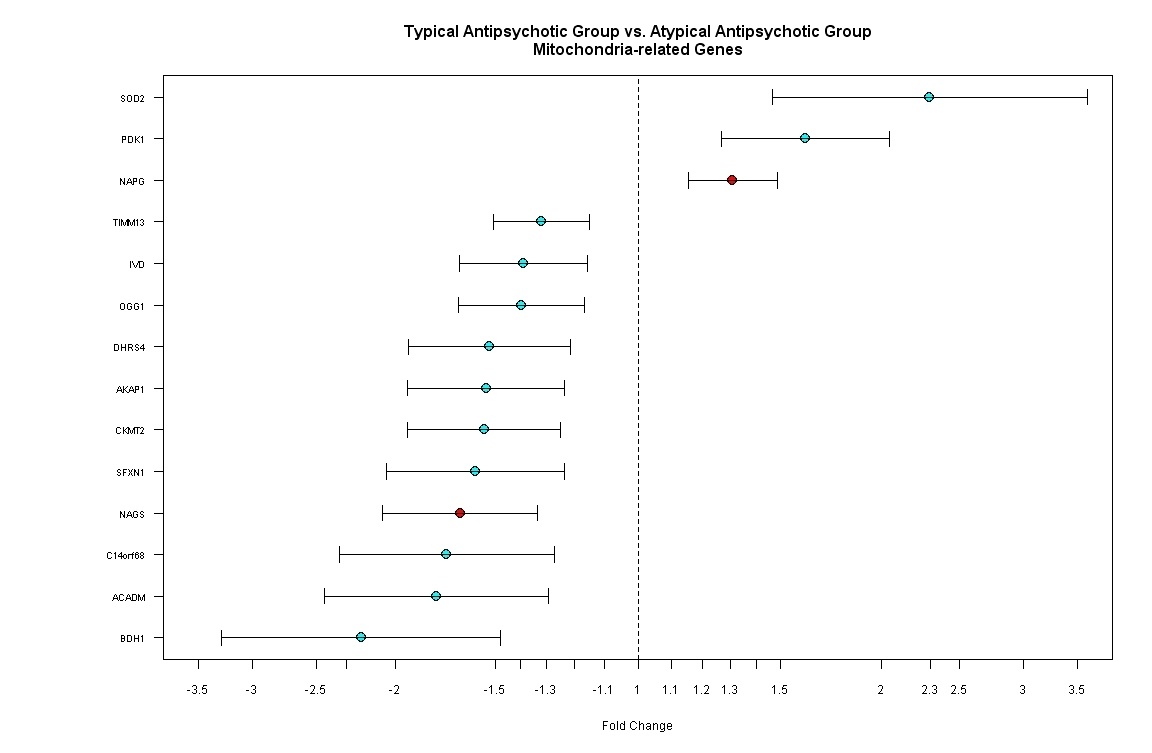

Supplement: Additional file 6 — Mitochondria genes. Genes associated with the mitochondrial function in typical AP group compared to atypical AP group. Each gene is plotted with fold change and 95% confidence intervals. Green: p < 0.001 and red: p < 0.0001 [file 1471-244X-9-57-S6.ZIP › additional_file6.jpg]
